# Supplementary figures and images for: Signaling by the integrated stress response kinase PKR is fine-tuned by dynamic clustering
Source: J Cell Biol. 2022 May 6;221(7):e202111100. doi: 10.1083/jcb.202111100 (PMC9086502; doi:10.1083/jcb.202111100)

p-eIF2α

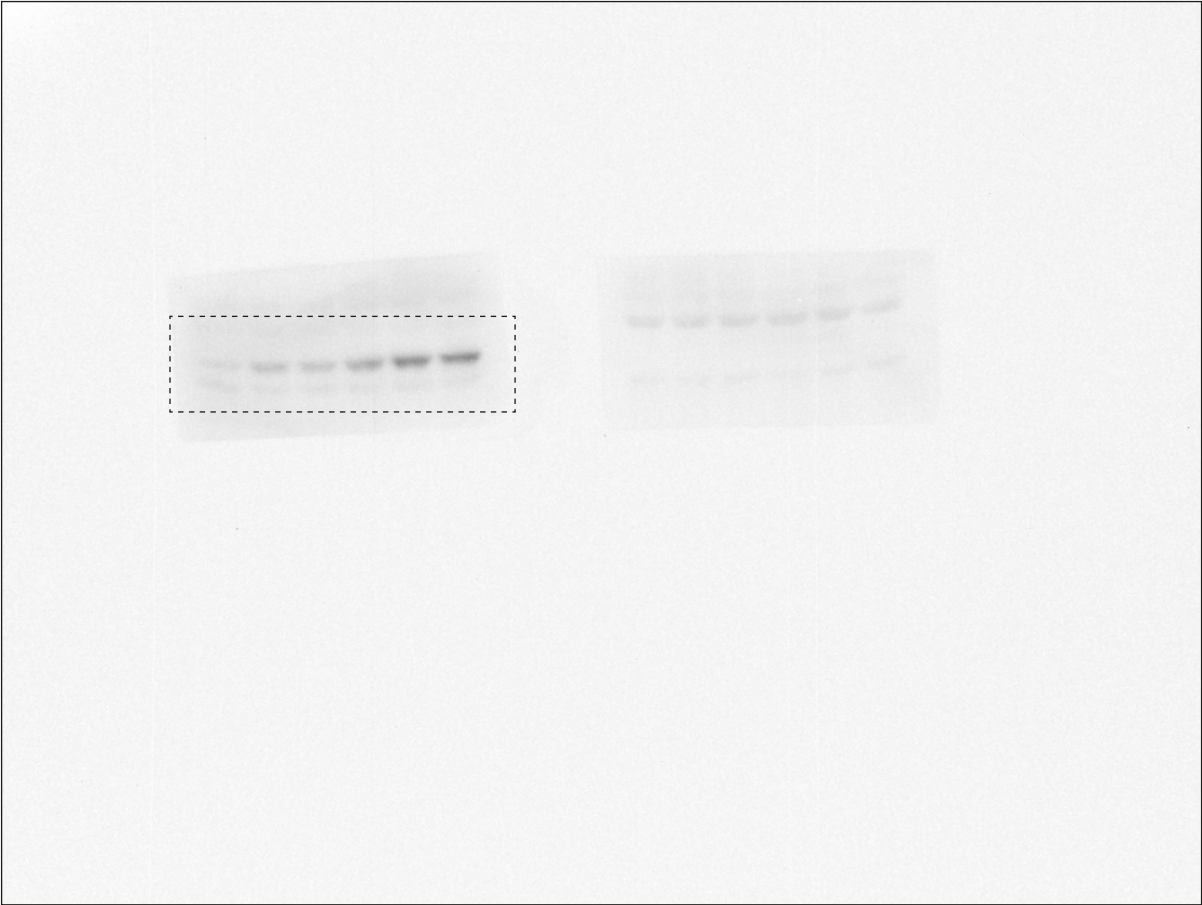

eIF2α

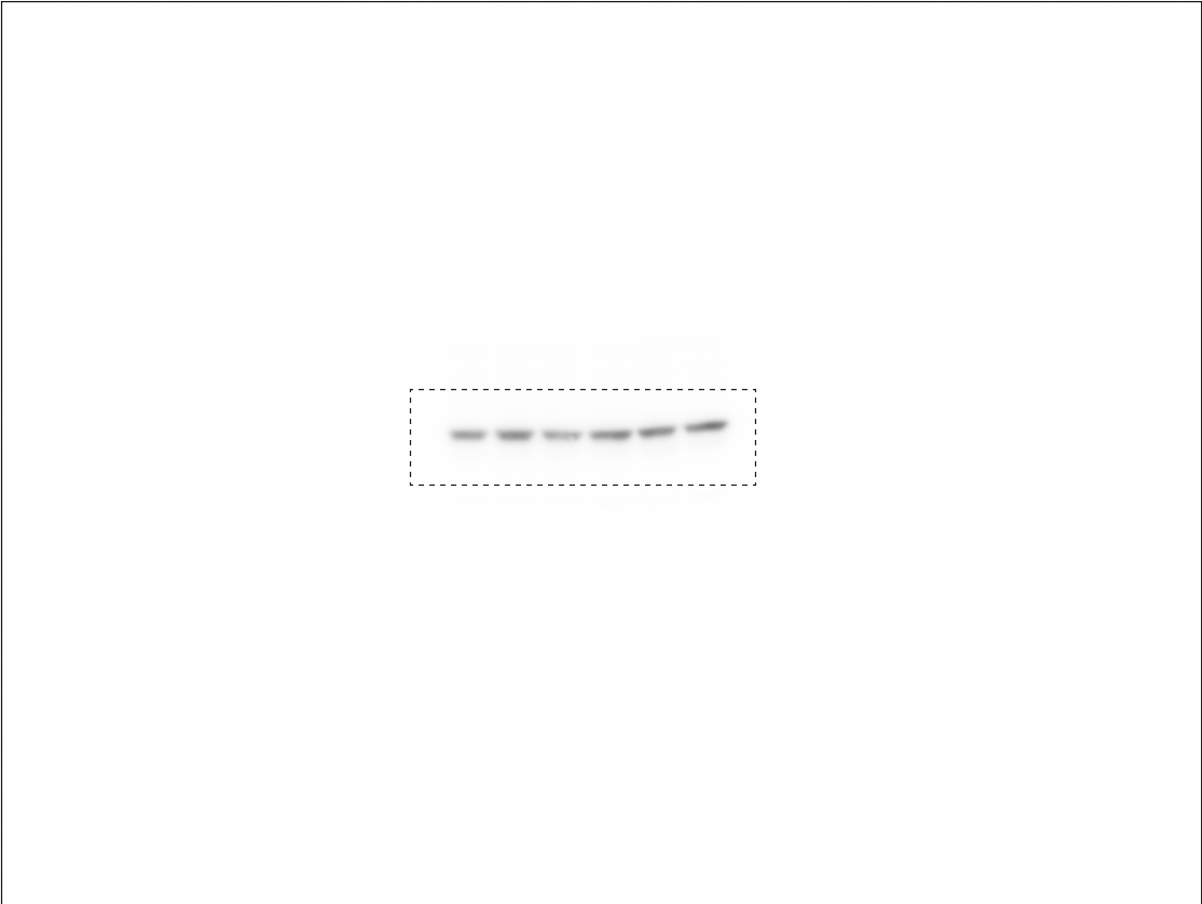

ponceau

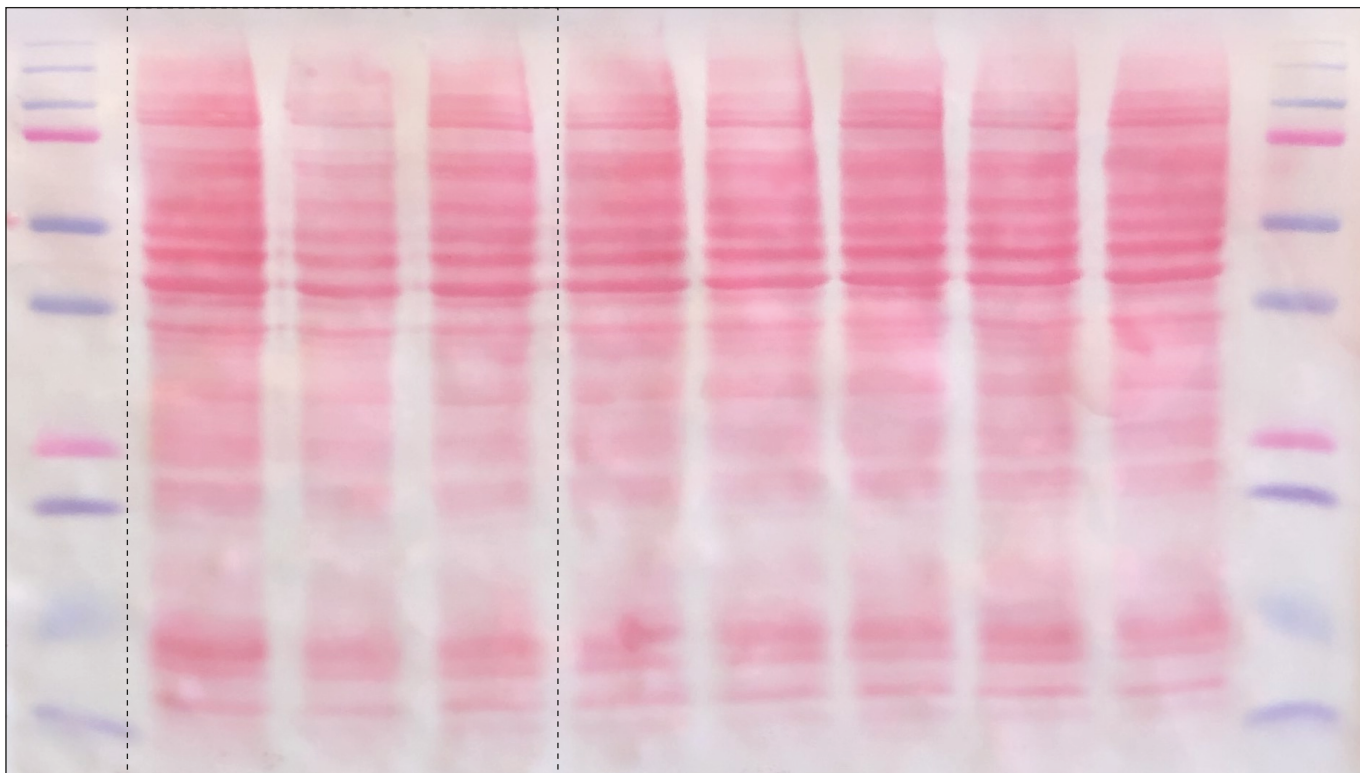

PMY

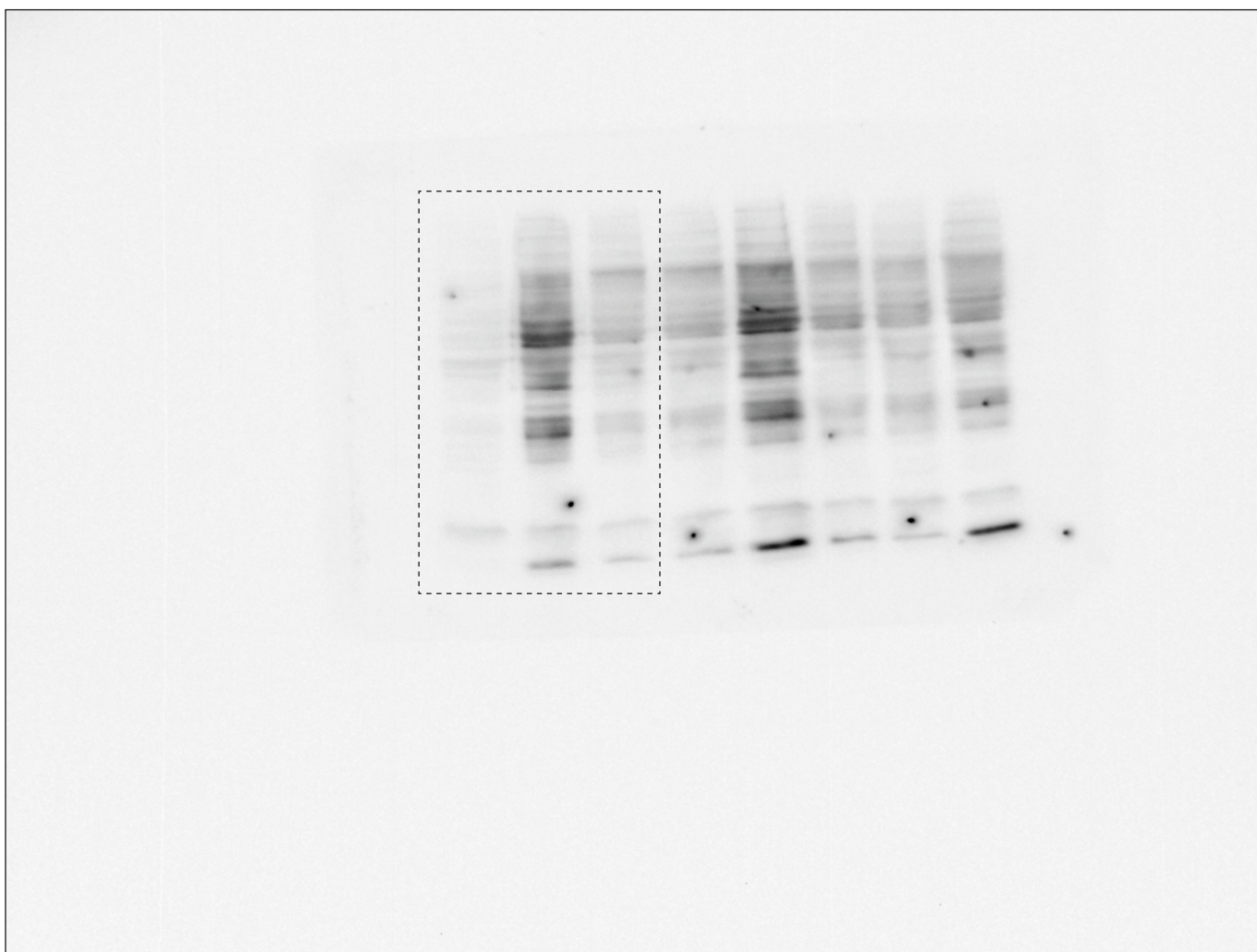

GADD34

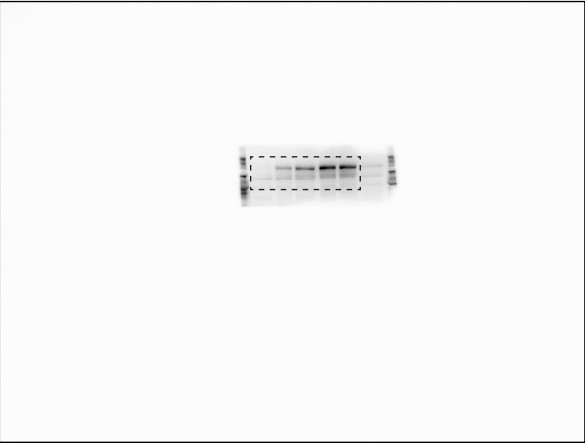

p-eIF2 $\alpha$ / eIF2 $\alpha$

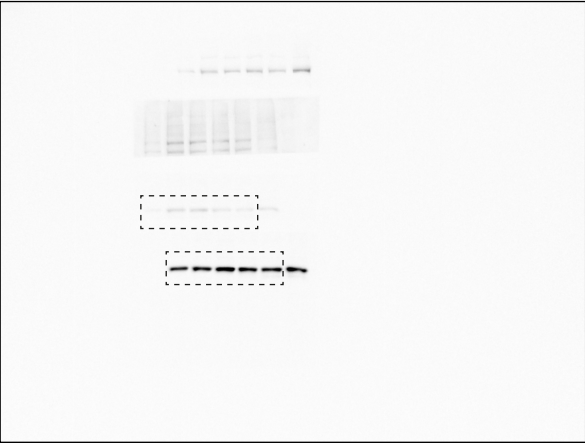

$\beta$ -actin

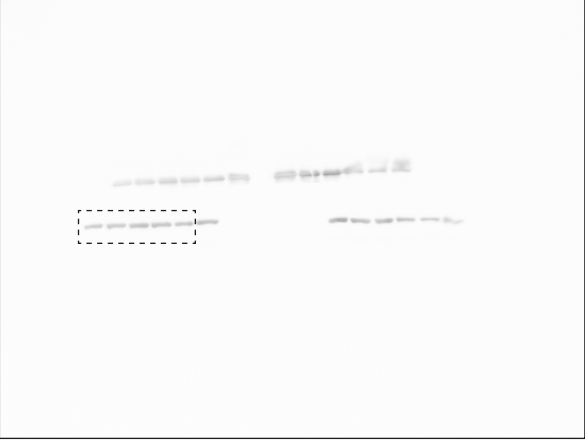

CHOP

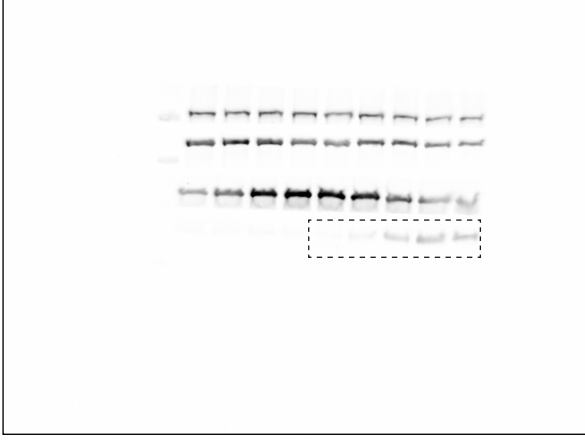

ATF4

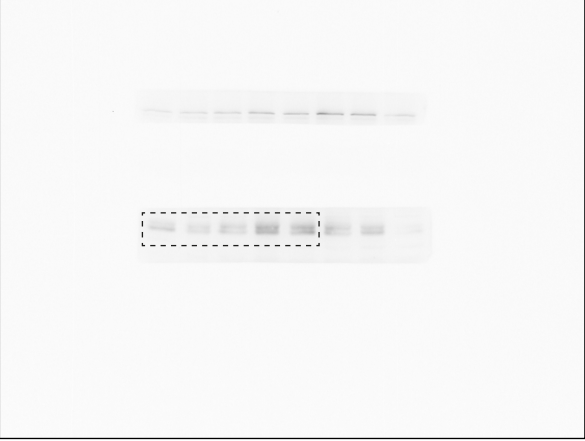

GAPDH

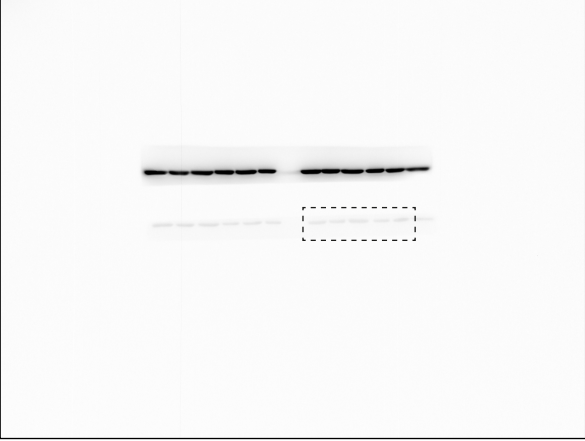

Supplement: SourceData F3 — is the source file for Fig. 3. [file JCB_202111100_SourceDataF3.pdf]

p-eIF2 $\alpha$

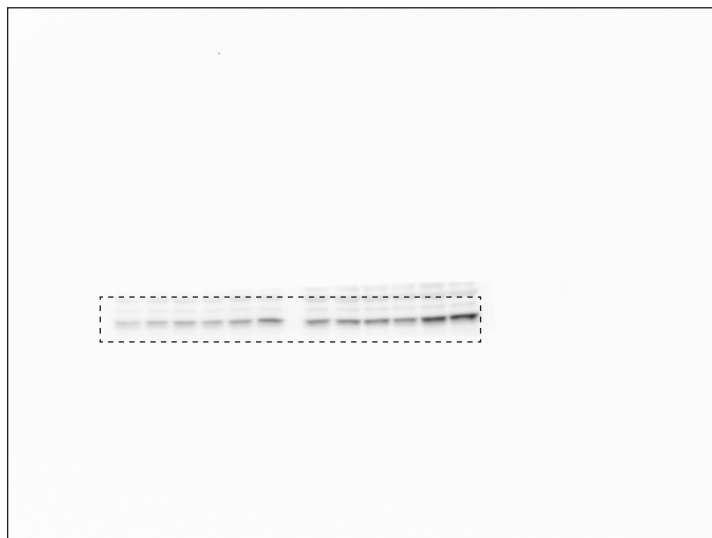

eIF2 $\alpha$

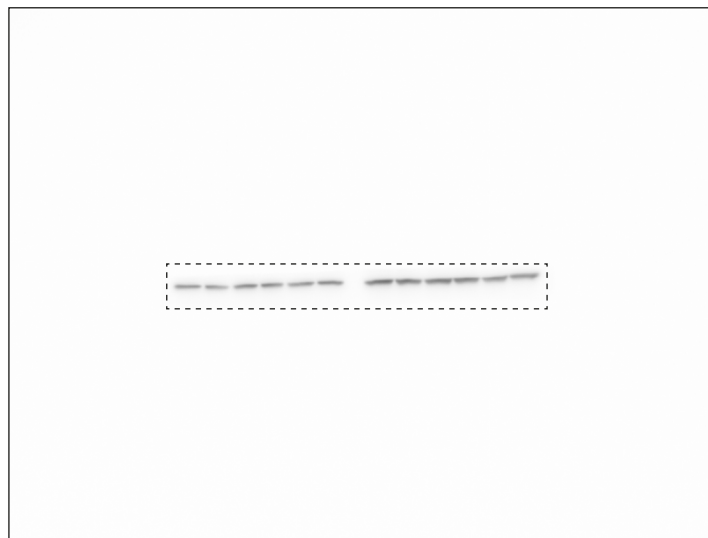

$\beta$ -actin

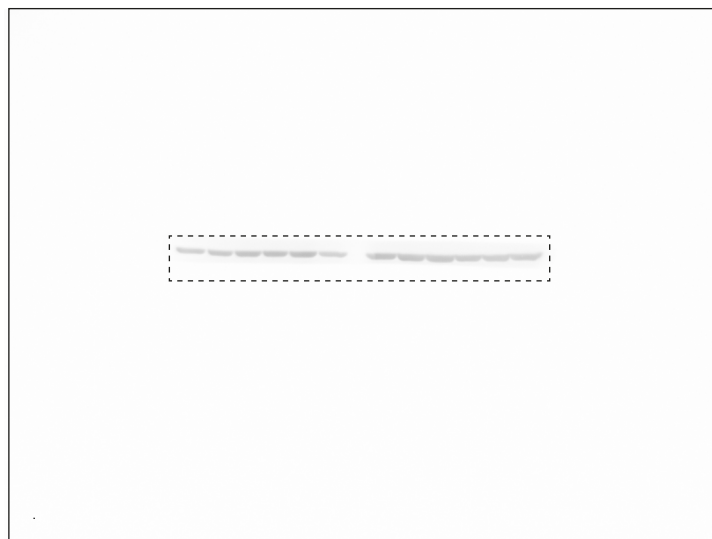

p-PKR

PKR

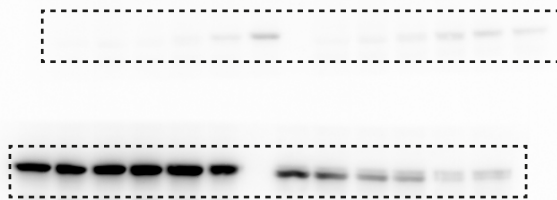

p-eIF2 $\alpha$

eIF2 $\alpha$

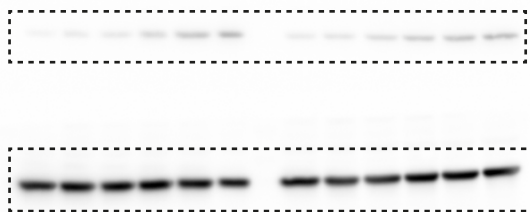

Supplement: SourceData F5 — is the source file for Fig. 5. [file JCB_202111100_SourceDataF5.pdf]

GAPDH

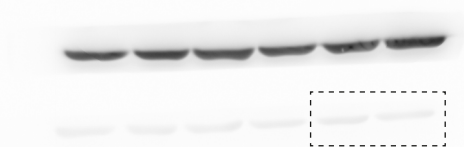

PKR

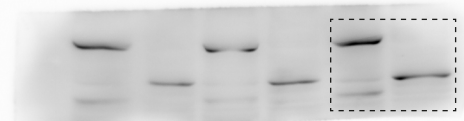

PKR

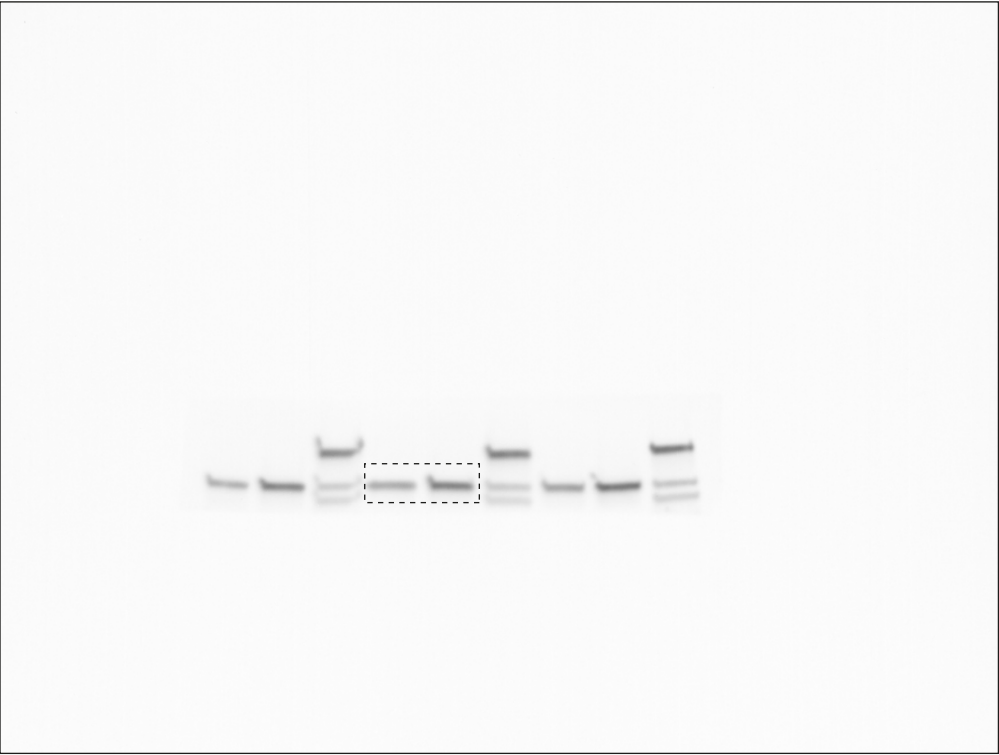

GAPDH

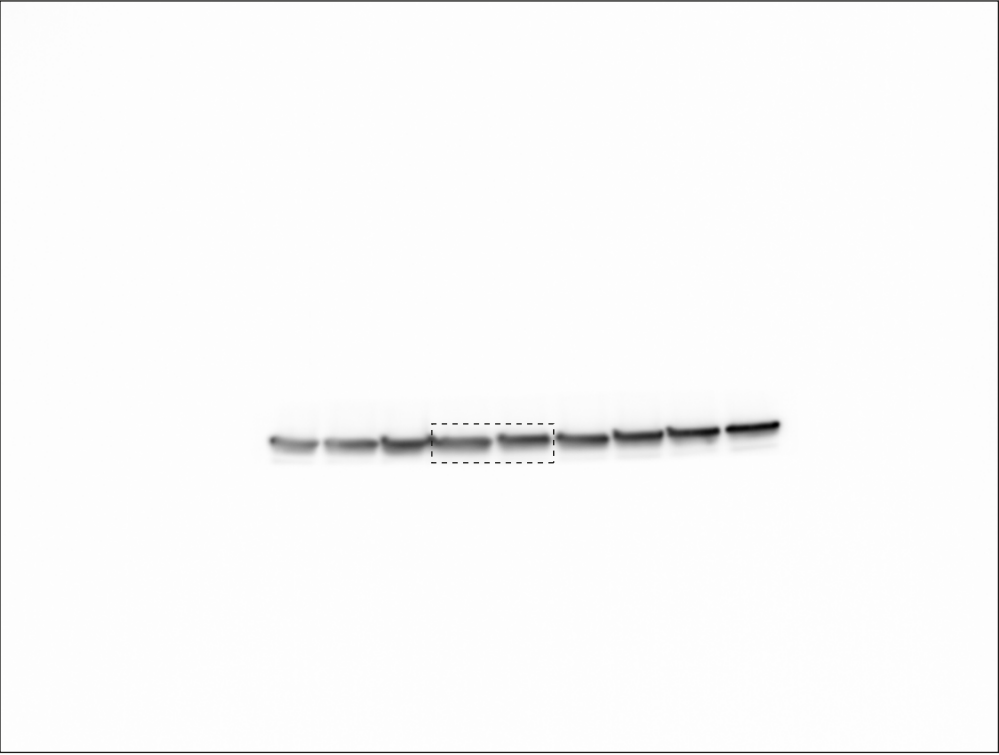

p-PKR

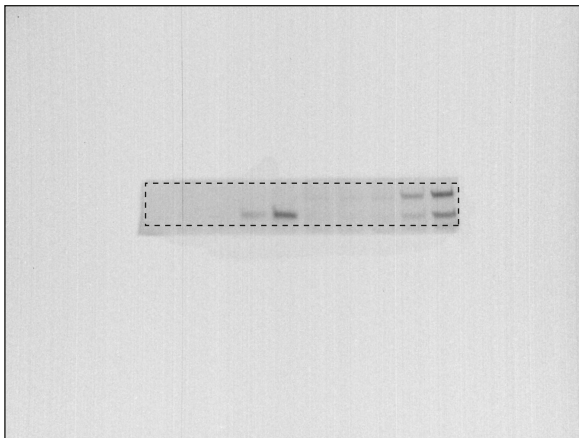

PKR

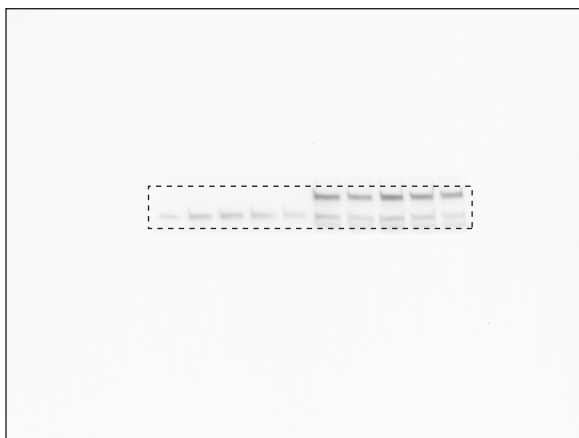

p-eIF2 $\alpha$

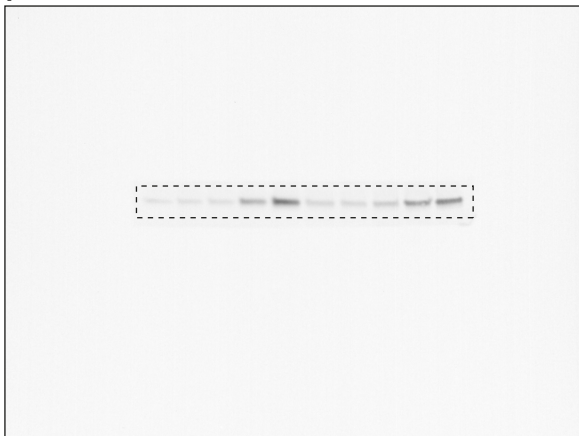

eIF2 $\alpha$

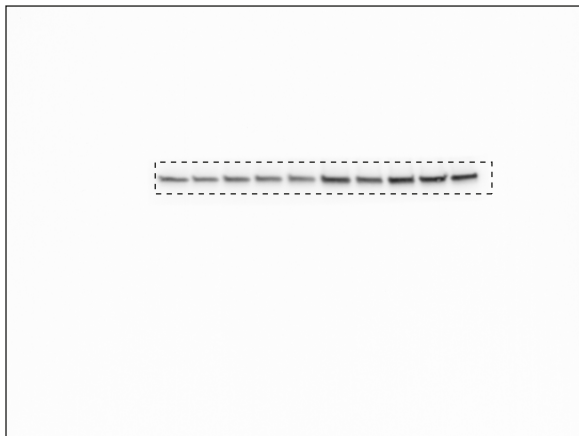

Supplement: SourceData FS1 — is the source file for Fig. S1. [file JCB_202111100_SourceDataFS1.pdf]

4E-T

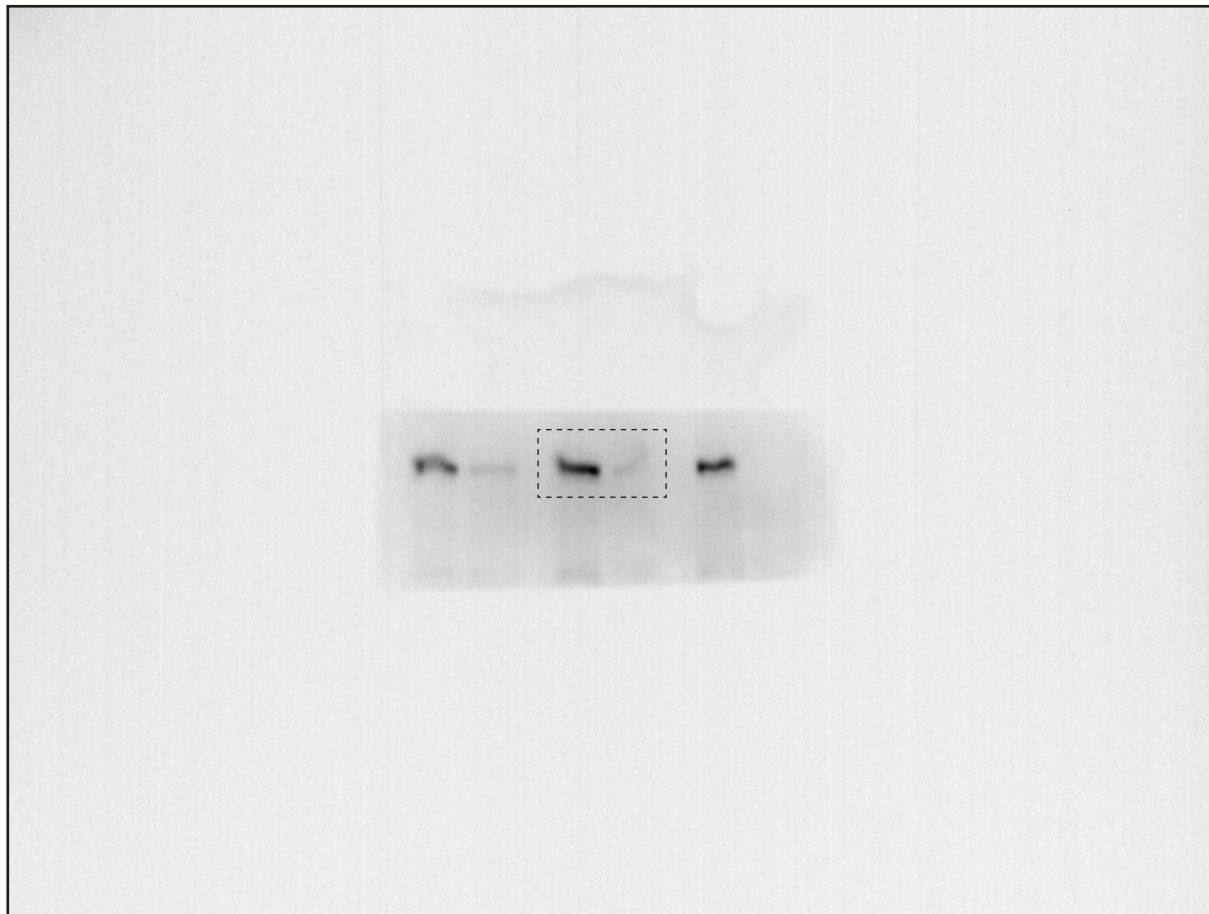

$\beta$ -actin

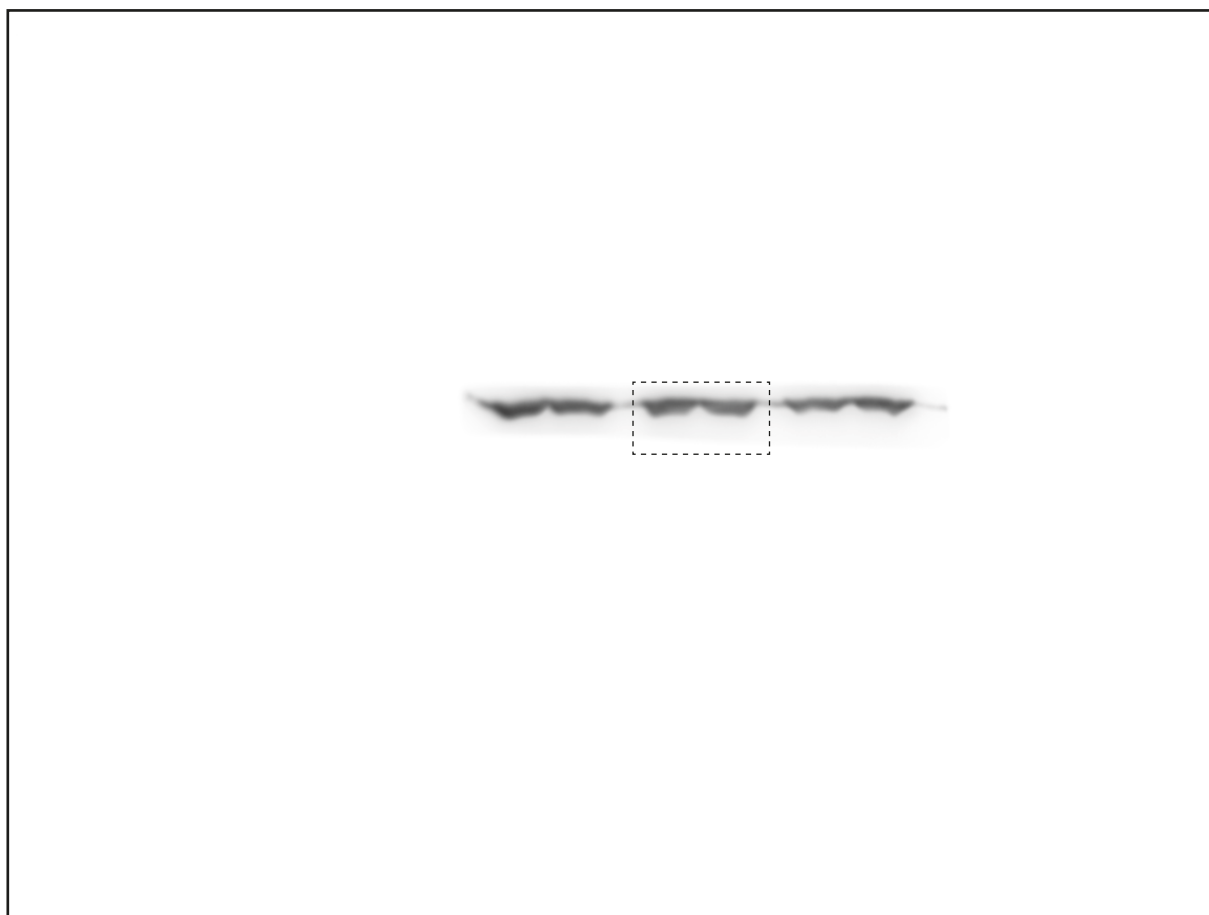

Supplement: SourceData FS2 — is the source file for Fig. S2. [file JCB_202111100_SourceDataFS2.pdf]

p-PKR

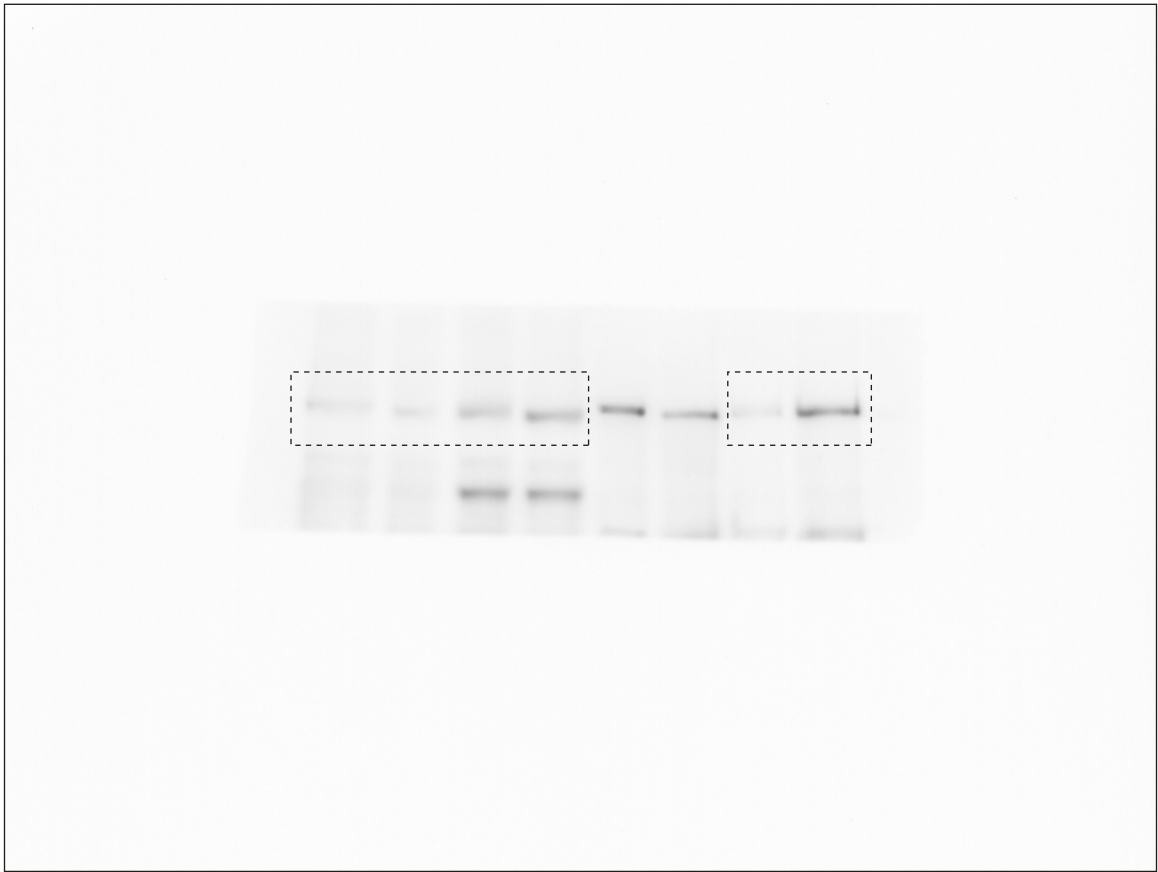

P-PKR

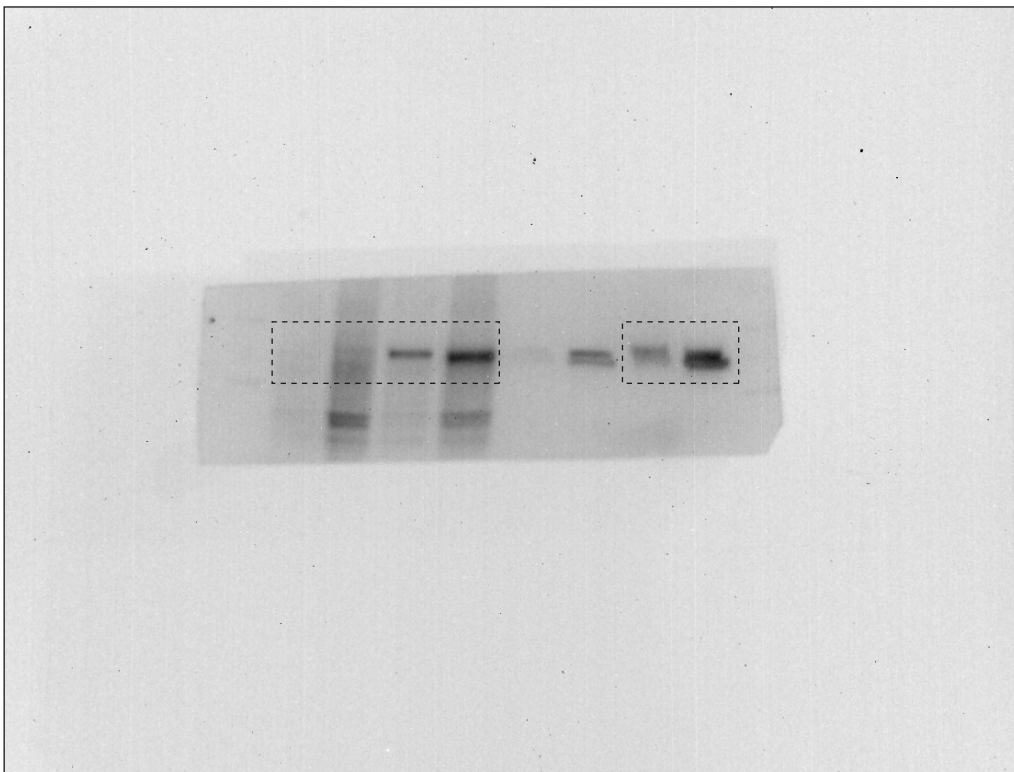

Flag

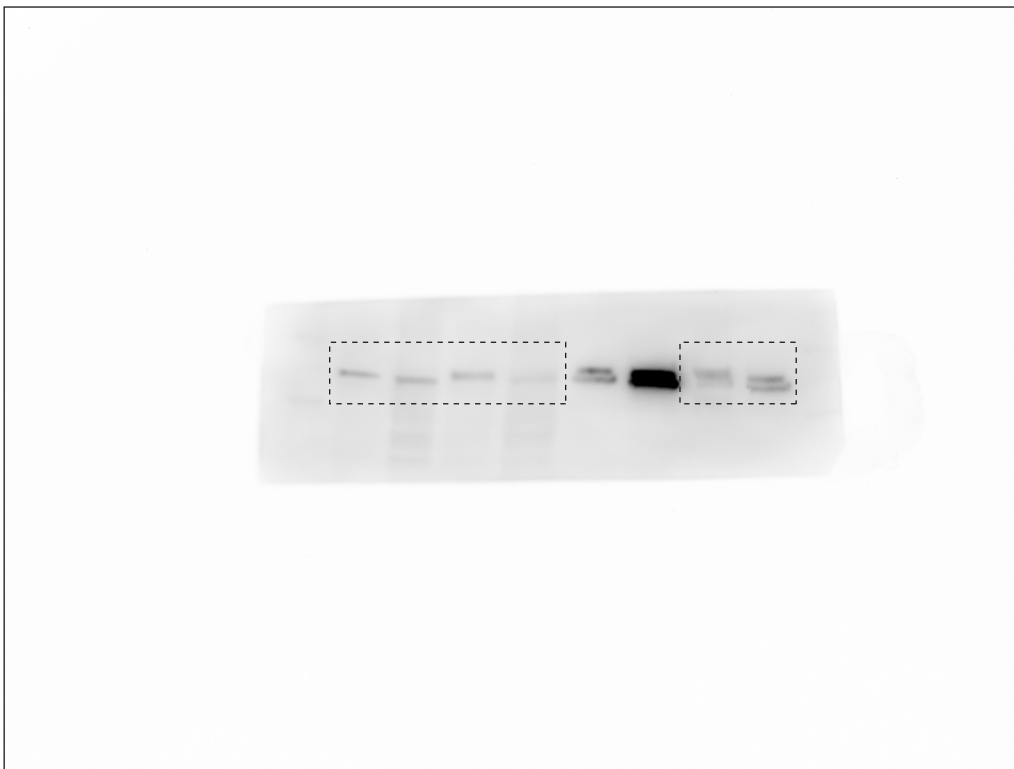

Supplement: SourceData FS5 — is the source file for Fig. S5. [file JCB_202111100_SourceDataFS5.pdf]
